# The crucial role of Erk2 in demyelinating inflammation in the central nervous system

**DOI:** 10.1186/s12974-016-0690-8

**Published:** 2016-09-05

**Authors:** Rentaro Okazaki, Toru Doi, Kentaro Hayakawa, Kazuhito Morioka, Osamu Imamura, Kunio Takishima, Makoto Hamanoue, Yasuhiro Sawada, Motoshi Nagao, Sakae Tanaka, Toru Ogata

**Affiliations:** 1 Department of Rehabilitation for Movement Functions, Research Institute, National Rehabilitation Center for Persons with Disabilities, 4-1 Namiki, Tokorozawa, Saitama 359-8555 Japan; 2 Department of Orthopaedic Surgery, The University of Tokyo, 3-7-1, Hongo, Bunkyo-ku, Tokyo, Japan; 3 Department of Biochemistry, National Defense Medical College, 3-1, Namiki, Tokorozawa, Saitama Japan; 4 Department of Physiology, Toho University, 5-21-16, Ohmorinishi, Ohta-ku, Tokyo, Japan

**Keywords:** Demyelination, MAPK, Chemokine

## Abstract

**Background:**

Brain inflammation is a crucial component of demyelinating diseases such as multiple sclerosis. Although the initiation of inflammatory processes by the production of cytokines and chemokines by immune cells is well characterized, the processes of inflammatory aggravation of demyelinating diseases remain obscure. Here, we examined the contribution of Erk2, one of the isoforms of the extracellular signal-regulated kinase, to demyelinating inflammation.

**Methods:**

We used the cuprizone-induced demyelinating mouse model. To examine the role of Erk2, we used Nestin-cre-driven Erk2-deficient mice. We also established primary culture of microglia or astrocytes in order to reveal the crosstalk between two cell types and to determine the downstream cascades of Erk2 in astrocytes.

**Results:**

First, we found that Erk is especially activated in astrocytes within the corpus callosum before the peak of demyelination (at 4 weeks after the start of cuprizone feeding). Then, we found that in our model, genetic ablation of Erk2 from neural cells markedly preserved myelin structure and motor function as measured by the rota-rod test. While the initial activation of microglia was not altered in Erk2-deficient mice, these mice showed reduced expression of inflammatory mediators at 3–4 model weeks. Furthermore, the subsequent inflammatory glial responses, characterized by accumulation of microglia and reactive astrocytes, were significantly attenuated in Erk2-deficient mice. These data indicate that Erk2 in astrocytes is involved in augmentation of inflammation and gliosis. We also found that activated, cultured microglia could induce Erk2 activation in cultured astrocytes and subsequent production of inflammatory mediators such as Ccl-2.

**Conclusions:**

Our results suggest that Erk2 activation in astrocytes plays a crucial role in aggravating demyelinating inflammation by inducing inflammatory mediators and gliosis. Thus, therapies targeting Erk2 function in glial cells may be a promising approach to the treatment of distinct demyelinating diseases.

## Background

The pathological processes of demyelinating diseases affecting the central nervous system (CNS), such as multiple sclerosis (MS), can be characterized as inflammation, demyelination, gliosis, and axonal degeneration [1, 2]. Within demyelinating lesions, oligodendrocytes are damaged by inflammation, which consists of both specific autoimmune responses involving T and B lymphocytes and non-specific noxious responses involving activated monocytes, microglia, and astrocytes [3, 4]. These reactions induce secretion of cell-toxic cytokines and the bioactive free radical nitric oxide, which cause oligodendrocyte cell death [5, 6]. Thus, regulation of such inflammatory reactions is now regarded as a possible therapeutic strategy for not only MS but also various other inflammatory diseases of the CNS [1].

Astrocytes are the most abundant cell type in the central nervous system and have various functions related to maintaining homeostasis in the brain [7]. However, in a pathological environment, astrocytes take on the reactive phenotype, which is characterized by increased cell processes, increased expression of GFAP and vimentin, and re-expression of nestin [8, 9]. Simultaneously, astrocytes produce various inflammation-related molecules and growth factors, resulting in gliosis [10]. Areas of gliosis were previously perceived as scar tissue resulting from disease processes. However, recent studies suggest that gliosis is actively involved in inflammatory reactions and that cellular functions within areas of gliosis are controlled by various signals [11].

Extracellular signal-regulated kinase (Erk) is a ubiquitous intracellular signal, and phosphorylation of Erk plays a pivotal role in a wide range of cellular activities, including survival, proliferation, and differentiation. The two isoforms of Erk, Erk1 (44 kDa) and Erk2 (42 kDa), share an 84 % identity at the amino acid level, and Erk2 can compensate for loss of most of the functions of Erk1 [12, 13]. On the other hand, because of lethality in Erk2 knockout mice, Erk2 is thought to have its own specific function [14, 15]. A study of the mechanism of gliosis found that Erk activity promotes the proliferation of cultured astrocytes [16]. However, the detailed function of Erk, especially of Erk2, in reactive astrocytes in areas of demyelinating inflammation is still elusive.

Here, we analyzed the role of Erk2 in the regulation of inflammation in the CNS using a model of demyelinating disease. In a cuprizone-induced demyelination model [17, 18], we observed activation of Erk in reactive astrocytes in the demyelinating lesion. We then ablated the function of Erk2 by either chemical inhibitors or genetic manipulation using nestin promoter-driven Cre expression and found amelioration of both the histological demyelination and the behavioral functional deficit. Further biochemical analyses both in vivo and in vitro revealed that the lack of Erk2 prevents astrocytes from producing cytokines and chemokines and attenuates the subsequent aggravation of inflammation. Our results reveal a crucial role for activation of astrocytic Erk2 in the progression of demyelinating inflammation.

## Methods

### Animals and reagents

To generate a conditional knockout of Erk2 in CNS, mice carrying loxP-Erk2 alleles (Erk2^f/f^) were crossed with transgenic mice expressing Cre recombinase under the control of the nestin promoter. Both Erk2^f/f^ mice [19] and Nestin-Cre^tg^ mice [20] were kindly provided by Dr. Takishima (National Defense Medical College, Saitama, Japan), Dr. Endo (Tokyo Metropolitan Institute of Gerontology, Tokyo, Japan), and Dr. R. Kageyama (Institute for Virus Research, Kyoto University, Kyoto, Japan). The transgenic Cre reporter mice (B6. Cg-Tg [CAG-floxed Neo-EGFP]) were provided by RIKEN Bioresource [21]. C57BL/6 mice and Wistar rats were obtained from Oriental Yeast (Tokyo, Japan). All animal experiments were approved by the ethical committee of the National Rehabilitation Center for Persons with Disabilities.

Cell-culture plastic ware was purchased from BD Biosciences (San Jose, CA, USA). Fetal bovine serum was purchased from MP biomedicals (Santa Ana, CA). Lipopolysaccharide (LPS; Escherichia coli O127:B8), the Mek inhibitor U0126, and cuprizone (bis-cyclohexanone oxaldihydrazone) were purchased from Sigma-Aldrich (St Louis, MO). The p38 inhibitor SB203580 and the NF-kB inhibitor SN50 were purchased from Merck Millipore (Billerica, MA). TO-PRO®-3 and ProLong® Gold were purchased from Invitrogen (Carlsbad, CA, USA).

### Cuprizone treatment

To produce demyelinated mice, 8- to 10-week-old (adult) C57BL/6 female mice were treated with a diet containing 0.2 % *w*/*w* cuprizone mixed into normal rodent chow for up to 6 weeks depending on the experimental condition [1, 17, 18]. After withdrawal of cuprizone from the diet, mice were returned to a normal diet [22, 23]. The animals were housed under standard laboratory conditions with food and water available ad libitum.

### Histological analysis

For tissue preparation and histological analysis, mice treated with cuprizone and untreated mice were perfused with phosphate-buffered saline (PBS) and 4 % paraformaldehyde in PBS and post-fixed for 24 h in the same fixative, followed by 24 h in 20 % sucrose/PBS and another 24 h in 30 % sucrose/PBS. Tissues were removed and embedded in O.C.T. compound for cryosection and stored at −80 °C for immunohistochemistry. Mouse brains were sectioned in 14-μm coronal serial sections onto slides using a Leica cryostat.

For immunohistochemical analyses, serial coronal sections were examined between levels −0.4 and −1.6 mm from bregma according to the Allen mouse brain atlas. Primary antibodies used were as follows: mouse anti-Erk1/2 (Santa-Cruz, Santa-Cruz, CA), rabbit anti-Erk1/2, rabbit anti-phospho-Erk1/2 (Cell Singaling Technology, Beverly, MA), rabbit anti-GFAP, mouse anti-GFAP, mouse anti-NeuN, rabbit anti-myelin basic protein (MBP; Millipore), anti-mouse CC1, rabbit mouse anti-Tuj1 (Calbiocchem), and rat anti-green fluorescent protein (GFP) (nacalai tesque, Kyoto, Japan). The cell density of Iba-1+ microglia and GFAP+ astrocytes or MBP positive area within 500 μm of the corpus callosum on either side was evaluated as relative immunoreactivity using a software package, WinROOF (Mitani-shoji, Japan). To examine the degree of demyelination, 14-μm serial cryosections of the corpus callosum between levels bregma −0.4 and bregma −1.6 mm according to Allen were stained with luxol fast blue (LFB) as previously described [24]. Demyelination was scored by two blinded investigators on a four-level scale running from 0 (normal myelination) to 3 (complete demyelination) [25].

### Quantitative real-time PCR

The animals were sacrificed under deep anesthesia and then perfused through the left ventricle with PBS. Then, the brain was quickly removed from the skull and the portion comprising the cortex, corpus callosum, striatum, and hippocampus (corresponding to bregma 0 to −2 mm) was isolated and stored in RNAlater reagent (Qiagen) until RNA extraction could be performed. Then, total RNA was extracted using the RNA extraction solution ISOGEN (Nippon Gene) and isolated using a standard kit (RNAeasy Mini kit; Qiagen), followed by reverse transcription (TaKaRa RNA PCR kit [AMV] Ver. 3.0; Takara). All quantitative PCR was performed on the 7500 Real-Time PCR System (Applied Biosystems) using the comparative Ct method. Power SYBR® Green (Applied Biosystems) was used to determine the relative Ct values. We calculated relative copy number of the targeted genes by comparison to the housekeeping genes GAPDH or beta2-microgloblin. Primers were designed using Biology Workbench (San Diego Supercomputer Center). The primer sequences are listed in Table 1.

**Table 1:** Primers used for RT-PCR

TNF-alpha (mouse)	Sense	5′-ACGGCATGGATCTCAAAGAC-3′
	Anti-sense	5′-GTGGGTGAGGAGCACGTAGT-3′
IL-1beta (mouse)	Sense	5′-CAGGCAGGCAGTATCACTCA-3′
	Anti-sense	5′-ATGAGTCACAGAGGATGGGC-3′
Ccl-2 (mouse)	Sense	5′-CCCAATGAGTAGGCTGGAGA-3′
	Anti-sense	5′-TCTGGACCCATTCCTTCTTG-3′
Ccl-3 (mouse)	Sense	5′-ATGAAGGTCTCCACCACTGC-3′
	Anti-sense	5′-GATGAATTGGCGTGGAATCT-3′
Ccl-5 (mouse)	Sense	5′-GTGCCCACGTCAAGGAGTAT -3′
	Anti-sense	5′-CACTTCTTCTCTGGGTTGGC-3′
Cxcl-10 (mouse)	Sense	5′-AAGTGCTGCCGTCATTTTCT-3′
	Anti-sense	5′-CAATGATCTCAACACGTGGG-3′
MBP (mouse)	Sense	5′-ACTCACACACGAGAACTACCCA-3′
	Anti-sense	5′-TGGTGTTCGAGGTGTCACAA-3′
MAG (mouse)	Sense	5′-GGTACATGGCGTCTGGTATTTCA-3′
	Anti-sense	5′-CCACTTGTGTGCGGGACTT-3′
TNF-alpha (rat)	Sense	5′-ACGGCATGGATCTCAAAGAC-3′
	Anti-sense	5′-GTGGGTGAGGAGCACGTAGT-3′
IL-1beta (rat)	Sense	5′-AAAAATGCCTCGTGCTGTCT-3′
	Anti-sense	5′-GGGATTTTGTCGTTGCTTGT-3′
Ccl-2 (rat)	Sense	5′-ATGCAGTTAATGCCCCACTC-3′
	Anti-sense	5′-TTCCTTATTGGGGTCAGCAC-3′
Ccl-3 (rat)	Sense	5′-CTTCTCCTATGGACGGCAAA-3′
	Anti-sense	5′-CGGTTTCTCTTGGTCAGGAA-3′
Ccl-5 (rat)	Sense	5′-ATATGGCTCGGACACCACTC-3′
	Anti-sense	5′-TGACAAAGACGACTGCAAGG-3′
Cxcl-10 (rat)	Sense	5′-CTTCCATGAACAGACGCTGA-3′
	Anti-sense	5′-TCTTGATGGCCTCAGATTCC-3′
GAPDH (rodent)	Sense	5′-TGCACCACCAACTGCTTAGC-3′
	Anti-sense	5′-GGATGCAGGGATGATGTTCT-3′

### Western blotting

The dissected brain tissues were homogenized in T-PER™ Mix (Roche, Indianapolis, IN) supplemented with protease inhibitor, 1 mM Na_3_VO_4_, 2 mM NaF, and EDTA, using a cell homogenizer (Wakenyaku, Kyoto, Japan) with 2-mm-diameter zirconia beads. The homogenate was then centrifuged at 15,000*g* for 20 min at 4 °C. The amount of protein in each sample was measured using a MicroBCA assay (Pierce, Rockford, IL). For processing cultured astrocytes, cells on 35-mm poly-d-lysine (PDL)-coated plates were lysed in M-PER™ mammalian protein extraction reagent (Pierce) containing a protease inhibitor (Roche), 1 mM Na_3_VO_4_, 2 mM NaF, and EDTA. Samples containing a standardized amount of protein (20 μg) were used for electrophoresis using e-PAGEL (ATTO, Tokyo, Japan). The proteins in the gel were then transferred onto an Immobilon-P membrane (Millipore, Bedford, MA). The blots were immunoreacted with anti-ERK1 (Zymed Laboratories, South San Francisco, CA), anti-ERK2 (Transduction Laboratories, Lexington, KY), anti-ERK1/2, anti-phospho-ERK2, anti-p38 mitogen-activated protein kinase (MAPK), anti-phospho-p38 MAPK, anti-c-Jun N-terminal kinase (JNK), anti-phospho-JNK, anti-Ikappa-B-alpha (all from Cell Signaling Technology), or anti-β-actin (Sigma) antibodies. Primary antibodies were used at 1:1000 dilution at 4 °C overnight in 5 % BSA TBS plus 0.01 % Tween 20 on a rotator. The secondary antibodies (anti-mouse or rabbit HRP conjugate; Promega, Fitchburg WI) were used at 1:15,000 dilution, and the immunoblots were developed using the ECL chemiluminescence system (Amersham Biosciences, Piscataway, NJ). The immunoblot signals were quantitatively analyzed with the Odyssey Fc Imaging System (LI-COR Biosciences, Lincoln, NE).

### Behavioral analysis

Motor function in the treated mice was assessed with the rota-rod test in a manner similar to that previously reported [26]. The mice were placed on a rotatable cylinder (Muromachi Kikai, Japan) that then accelerated from 0.5 to 50 rpm over 180 s. The time when the mice fell off or clung tightly to the cylinder for two rotations was measured as the score. The trial was repeated 10 times and the best five scores were averaged. Mice were pre-trained in the same protocol for 2 weeks prior to starting cuprizone feeding.

### Cell culture

The primary astrocyte and microglia cultures were established as follows. Mixed glial cultures from E19 Wistar rats, Erk2^f/f^ mice, and Erk2 cKO mice were established from neonatal cortices. Cells were cultured in DMEM High Glucose (Wako) supplemented with 10 % FBS, penicillin, and streptomycin. The culture medium was replaced with fresh medium 24 h after the initial preparation and every 3 days thereafter. After 1 week, microglia were obtained by mechanical shaking and were then were seeded onto PDL-coated dishes to obtain conditioned medium. After further shaking, the remaining astrocytes were replated on new PDL-coated dishes for further analysis. Cells were monitored for purity by immunofluorescence and were routinely found to be >98 % positive for GFAP. Microglia-conditioned medium was obtained by treating microglia with DMEM containing 0.1 μg/mL LPS (stimCM) or vehicle (non-stimCM) for 3 h. To remove LPS from the medium, the media were switched to fresh, serum-free DMEM and cultured for an additional 21 h. The obtained microglia-conditioned medium was stocked at −20 °C. Astrocyte cultures were starved without serum for 3 h before treatment with either stimCM or non-stimCM for 24 h. In the proliferation assay, BrdU was added to the medium 6 h before fixation.

### Statistical analysis

Statistical analysis was performed using SPSS ver. 21 software. Student’s *t* tests were used for the statistical analysis of differences in the RT-PCR data between two groups. Statistical analysis of rota-rod test scores was by two-way repeated ANOVA, followed by the post hoc Bonferroni’s rho test. To quantify the weekly demyelination scores, non-parametric analysis was performed using the Kruskal-Wallis test, followed by the Mann-Whitney test. All data are presented as mean ± SD.

## Results

### Erk activation in astrocytes during cuprizone intoxication

**Fig. 1:**
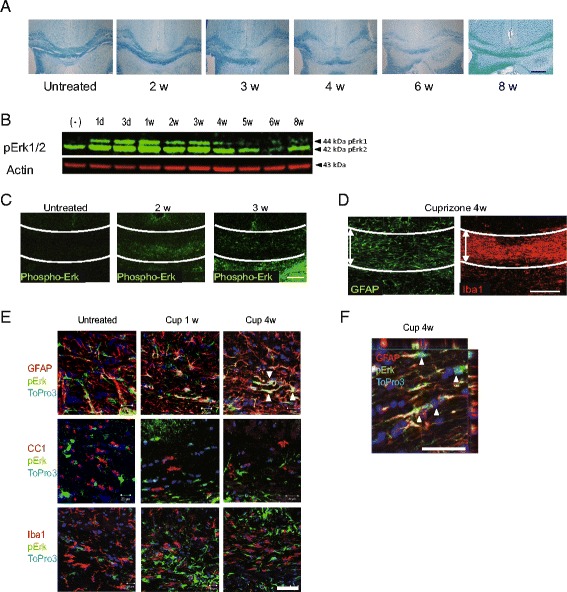
Phosphorylation of Erk1/2 during cuprizone-induced demyelination. **a** Progressive demyelination observed in the corpus callosum as shown by reduced luxol fast blue (LFB) staining. *d, W* days or weeks of cuprizone feeding. **b** Phosphorylation of Erk1 and Erk2 shown by protein analysis of the corpus callosum. Three independent experiments showed the same trend. The representative data is shown. **c** Phosphorylation of Erk1 and Erk2 shown by immunohistochemistry of the region. **d** At the fourth model week, when demyelination occurs, gliosis is observed as shown by increased immunoreactivity for GFAP and Iba-1. Lines in **d** and **e** indicate the boundaries of the corpus callosum; *white, double-headed arrows* indicate the area of the corpus callosum. **e**, **f** Multiple immunostaining indicates that a phosphorylated form of Erk1/2 becomes prominent and colocalizes with GFAP-positive cells (**e**, **f** *arrowhead*) but only occasionally with CC1- or Iba-1-positive cells. *Cup* cuprizone treatment duration; TO-PRO®-3, a nuclear stain; *scale bars*: **a**, **c**, and **d,** 100 μm; **e** and **f**, 50 μm

We first examined a 6-week serial time course of the cuprizone feeding model, focusing on the corpus callosum, where demyelination reproducibly occurs. Demyelination in the corpus callosum, characterized by reduced LFB staining, became prominent after 3 weeks of cuprizone treatment, and extensive demyelination was observed between 4 and 6 weeks. Remyelination was observed at 8 weeks, 2 weeks after the mice had been returned to a cuprizone-free diet (Fig. 1a). To investigate the involvement of Erk1/2 signaling pathways in the demyelinating process, we performed western blotting analyses of corpus callosum. Figure 1b shows early activation of Erk1/2 during cuprizone treatment (Fig. 1b). In accordance with these results, histological specimens obtained from cuprizone-treated mice showed increased immunoreactivity for phospho-Erk1/2 in the corpus callosum compared to untreated mice (Fig. 1c).

In addition to the changes observed in myelin structure, accumulation of Iba-1-positive microglia/macrophages and GFAP-positive astrocytes was also seen in demyelinated corpus callosum at 4 weeks (Fig. 1d). These glial cell accumulations coincided with a reduction of CC1-positive mature oligodendrocytes in the corpus callosum (Fig. 1e). We then evaluated the cell specificity of phospho-Erk1/2 in demyelinating lesions. Notably, we detected phospho-Erk1/2 mainly in GFAP-positive astrocytes and occasionally in Iba-1-positive microglia and CC1-positive oligodendrocytes (Fig. 1e). Phospho-Erk1/2 was located in both nucleus and cytoplasm (Fig. 1f). Taken together, our findings suggest that Erk1/2 phosphorylation was induced in astrocytes located in gliotic lesions during cuprizone-induced demyelination.

### Selective deletion of Erk2 in neural cells including astrocytes but not in microglia

**Fig. 2:**
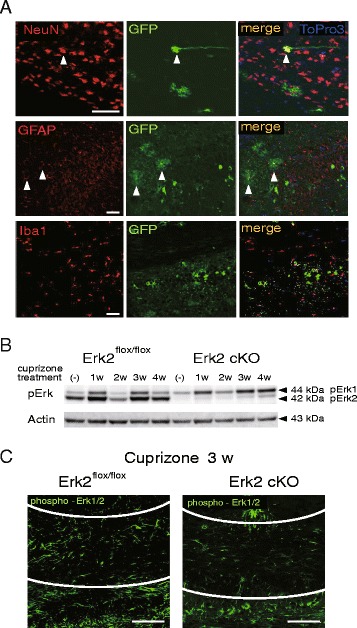
Deletion of Erk2 in neural cell population. **a** Immunohistochemical staining of samples of cortex taken from adult Nestin-Cre^tg^: CAG-floxed Neo-EGFP mice confirms that recombination occurs in neurons (NeuN+) and astrocytes (GFAP+) (**a**, *arrowhead*) but not in microglia (Iba-1+). **b**, **c** Nestin-Cre^tg^: Erk2^flox/flox^ (Erk2 cKO) mice treated with cuprizone. Western blotting of phospho-Erk shows selective deletion of Erk2 (**b**). Immunoreactivity of phospho-Erk is also reduced in corpus callosum in cuprizone-treated Erk2 cKO mice (**c**). Note that antibody against phospho-Erk reacts with both phospho-Erk1 and phospho-Erk2. The lines in **c** indicate the boundary of the corpus callosum. *Scale bars*: **a**, 50 μm; **c**, 100 μm

To investigate the role of Erk1/2 signaling in astrocytes, we attempted genetic manipulation of the signaling pathway in the demyelination model. Among two isoforms of Erk, Erk1 and Erk2, we focused on Erk2 because Erk2 has specific roles that are not compensated by Erk1. Because the Erk2 knockout is embryonically lethal [19], we employed Erk2 conditional knockout (cKO) mice, by cross-breeding Erk2-floxed mice and Nestin-Cre^tg^ mice. In these mutant mice, Erk2 is abrogated in neuronal and glial cells derived from nestin-positive neural stem cells [14]. In order to confirm Erk2 deletion, we used Cre reporter mice (B6. Cg-Tg [CAG-floxed Neo-EGFP]) crossbred with Nestin-Cre^tg^ mice, in which the fluorescent reporter protein EGFP was expressed in progenies of cells in which Cre recombination had occurred. In these transgenic mice, after 1 week of cuprizone feeding, we observed EGFP in neurons and astrocytes, but not in microglia, which are developmentally derived from bone marrow (Fig. 2a).

We then evaluated the efficacy of Erk2 ablation after cuprizone intoxication. Western blotting analysis of the corpus callosum showed adequate Erk2 ablation in Erk2 cKO mice (Fig. 2b). Immunohistologically, after 3 weeks of cuprizone feeding, Erk1/2 phosphorylation in the corpus callosum was found to be lessened in Erk2 cKO mice (Fig. 2c) relative to the Erk2-floxed background strain. These results indicate that cuprizone-induced Erk2 activation in the corpus callosum was prominently reduced in Erk2 cKO mice.

### Erk2 cKO mice showed preserved white matter and better motor function in the cuprizone model

**Fig. 3:**
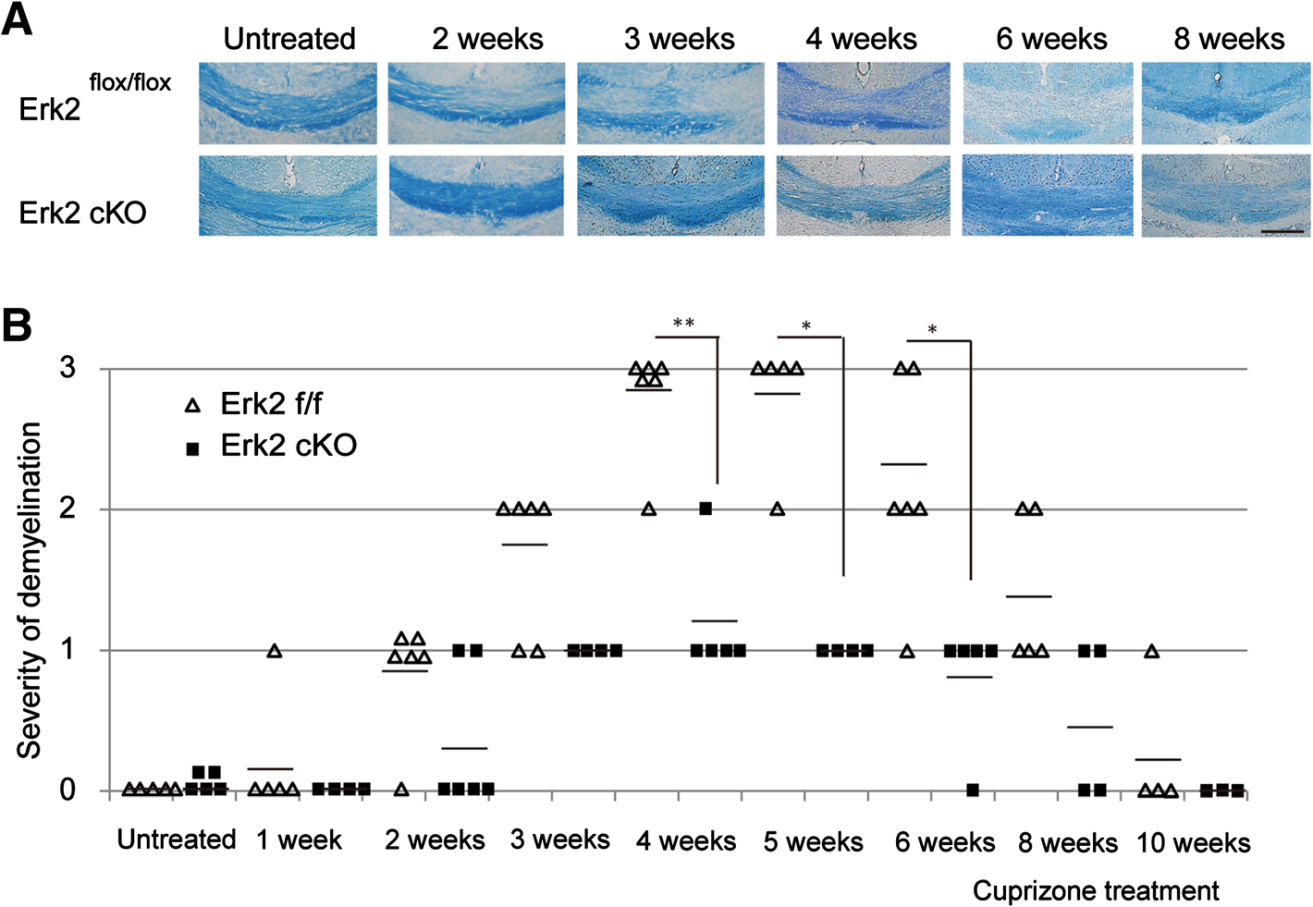
Erk2 cKO mice are less sensitive to cuprizone-induced demyelination. Erk2 cKO or Erk2 ^flox/flox^ mice were fed with cuprizone for 1 to 10 weeks before histological examination. **a** Representative LFB staining shows ameliorated demyelination in Erk2 cKO mice compared with Erk2 ^flox/flox^ mice. *Scale bar*, 50 μm. **b** The severity of demyelination compared between Erk2 cKO and Erk2 ^flox/flox^ mice using a demyelination score. Statistical differences are observed at 4, 5, and 6 weeks of cuprizone feeding (*n* = 3 to 5; Mann-Whitney *U* test at each time point; *p < 0.05; **p < 0.01). *Individual symbols* represent individual animals

**Fig. 4:**
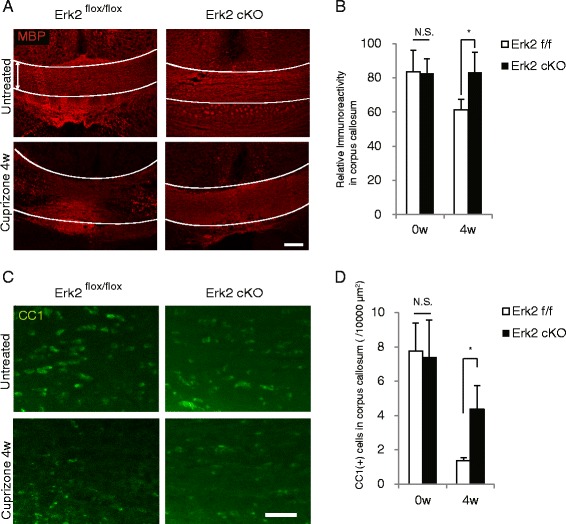
Mature oligodendrocytes were preserved in Erk2 cKO mice after cuprizone-induced demyelination. Histological samples of corpus callosum were obtained from either Erk2 cKO or Erk2 ^flox/flox^ mice before and 4 weeks after the start of cuprizone feeding for either MBP staining (**a**) or CC1 staining (**c**). In Erk2 ^flox/flox^ mice, immunoreactivity for MBP is decreased at 4 weeks compared to untreated (**a** left), while such changes are less prominent in Erk2 cKO mice (**a** right). **b** The immunoreactivity for MBP is higher in Erk2 cKO than Erk2 ^flox/flox^ at 4 weeks of cuprizone feeding. **c**, **d** The number of CC1 positive cells are reduced after cuprizone treatment in Erk2 ^flox/flox^, while there are more CC1 positive cell remaining in corpus callosum of Erk2 cKO. (**b**, **d**: *n* = 3; Student’s *t* test; *, *p* < 0.05). *Scale bars*: **a**, 100 μm; **c**, 50 μm

To assess the effects of Erk2 deletion on cuprizone-induced demyelination, LFB-stained sections from Erk2 ^flox/flox^ and Erk2 cKO mice were scored on a scale of 0 (normal myelination) to 3 (complete demyelination). Erk2 cKO mice showed significant reductions in demyelination after 4, 5, and 6 weeks of cuprizone treatment (Fig. 3a, b). Immunohistological analysis using MBP staining also showed the myelin protein reduction after cuprizone treatment in Erk2 ^flox/flox^, while samples from Erk2 cKO showed relative preservation of the myelin protein even after cuprizone treatment (Fig. 4a, b). The preservation of myelin proteins in the tissue indicates two possibilities: the delay in removal of degenerated myelin or preservation of mature oligodendrocytes. To test these possibilities, we examined the number of CC1-positive mature oligodendrocytes in the demyelination model. We found more CC1-positive cells were observed in Erk2 cKO mice than Erk2 ^flox/flox^ after cuprizone treatment (Fig. 4c, d). This result indicates the ablation of Erk2 signaling leads to amelioration of oligodendrocytes loss in the demyelinating model.

**Fig. 5:**
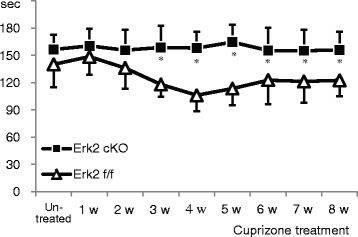
Better motor function of Erk2 cKO mice in the demyelinating model. Erk2 cKO or Erk2 ^flox/flox^ mice were fed with cuprizone for 8 weeks with weekly rota-rod test evaluation. Rota-rod test reveals that Erk2 cKO mice are able to remain on the cylinder longer than Erk2 ^flox/flox^ mice at 3 to 8 weeks of cuprizone feeding (*n* = 8; two-way repeated ANOVA followed by post hoc Bonferroni test; *p < 0.05)

Further, to test the effect of Erk2 deletion on motor function, we assessed rota-rod test performances in Erk2 ^flox/flox^ and Erk2 cKO mice after demyelination. We found no significant group differences at the beginning of cuprizone feeding (and after the 2-week training period). In Erk2 ^flox/flox^ mice, the rota-rod scores gradually decreased after the start of cuprizone feeding (35 % reduction at 4 weeks), then showed an improving trend after 5 weeks. However, the Erk2 cKO mice maintained their scores during cuprizone feeding and showed a significant superiority to the Erk2 ^flox/flox^ mice after 3 weeks (Fig. 5). These findings suggest that Erk2 deletion ameliorates demyelination and motor dysfunction in the cuprizone demyelination model.

### Lack of Erk2 did not alter the initial response to cuprizone feeding

**Fig. 6:**
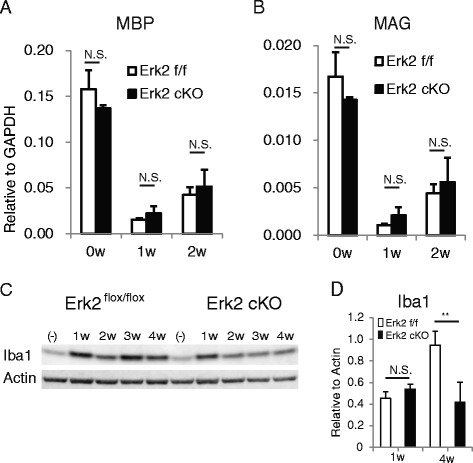
Erk2 cKO and Erk2 ^flox/flox^ mice manifest similar reactions in the early phase of the cuprizone model. Damage to oligodendrocytes is quantified as decreases in the level of expression of the myelin-related molecules MBP (**a**) and MAG (**b**). No significant differences are seen between Erk2 cKO and Erk2 ^flox/flox^ after cuprizone feeding. *n* = 4; Student’s *t* test. **c** Activation of microglia analyzed by immunoblotting of Iba-1, which reveals differences between Erk2 cKO and Erk2 ^flox/flox^ at 4 weeks but not at 1 week of cuprizone feeding. **d** quantitative analysis: *n* = 4; Student’s *t* test; **p < 0.01

In cuprizone-induced demyelination, the toxin directly induces oligodendrocyte cell damage. The cell damage induces the microglial activation observed at 1 week of cuprizone feeding, which leads to augmented inflammatory reactions involving both microglia and astrocytes at later time points (most prominently at 3–4 weeks) [17, 23]. To test the possibility that Erk2 deletion exerts its effects before astrocyte activation, we examined the samples at early time points. In the early phase of cuprizone intoxication, a reduction in expression of the messenger RNA (mRNA) of myelin-related molecules correlated with cuprizone-induced oligodendrocyte cell damage [1, 6]. We assessed expression of the mRNA for myelin basic protein (MBP) and myelin-associated glycoprotein (MAG) during cuprizone intoxication. Quantitative RT-PCR showed significant reduction of MBP and MAG levels after 1 week of exposure to cuprizone. However, no significant difference was found between Erk2 cKO and Erk2 ^flox/flox^ mice (Fig. 6a, b). As for the activation of microglia, western blot analysis showed no significant differences between Erk2 cKO and Erk2 ^flox/flox^ mice in the elevations of Iba-1 protein at 1 week of cuprizone exposure, while significant reduction was seen in Erk2 cKO mice at 4 weeks (Fig. 6c, d). These results indicate that conditional Erk2 deletion in the CNS altered neither the initial oligodendrocyte damage nor the microglia activation in the early phase of cuprizone intoxication.

### Erk2 deletion attenuated gliosis and expression of mediators of inflammation

**Fig. 7:**
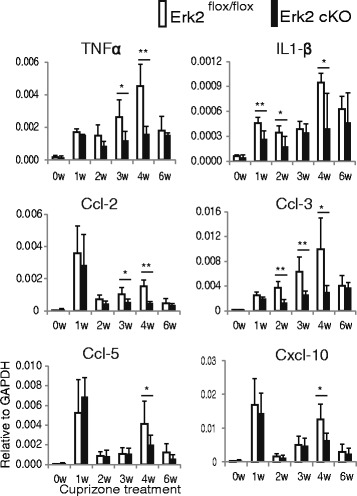
Cuprizone-induced inflammation is attenuated in Erk2 cKO mice. Erk2 cKO or Erk2 ^flox/flox^ mice were fed with cuprizone for 0, 1, 2, 3, 4, and 6 weeks before mRNA sampling. Cytokine and chemokine mRNA is quantitated as amount relative to GAPDH mRNA. Note that at 4 weeks of cuprizone feeding, all inflammatory molecules tested are higher in Erk2 ^flox/flox^ mice than in Erk2 cKO mice (*n* = 4; Student’s *t* test; *p < 0.05; **p < 0.01)

The early microglia activation seen in studies of demyelinating disease is followed by elevated expression of inflammatory mediators [17, 27]. Therefore, we first assayed these mediators in demyelinating corpus callosum in our model. Quantitative RT-PCR analysis of the corpus callosum showed that expression of the mRNA for pro-inflammatory cytokines (TNF-alpha and IL-1beta) and chemokines (Ccl-2, Ccl-3, Ccl-5, and Cxcl-10) increased during cuprizone intoxication. In the control (Erk2 ^flox/flox^) mice, the data shows that some of the chemokines (Ccl-2, Ccl-5, and Cxcl-10) had expression peaks at both 1 and 4 weeks, while others had a single peak at 4 weeks of cuprizone treatment. On the other hand, in Erk2 cKO mice, only IL-1beta still showed the peak at 1 week after Erk2 knockout, while at 4 weeks all of the inflammatory mediators were reduced (Fig. 7).

**Fig. 8:**
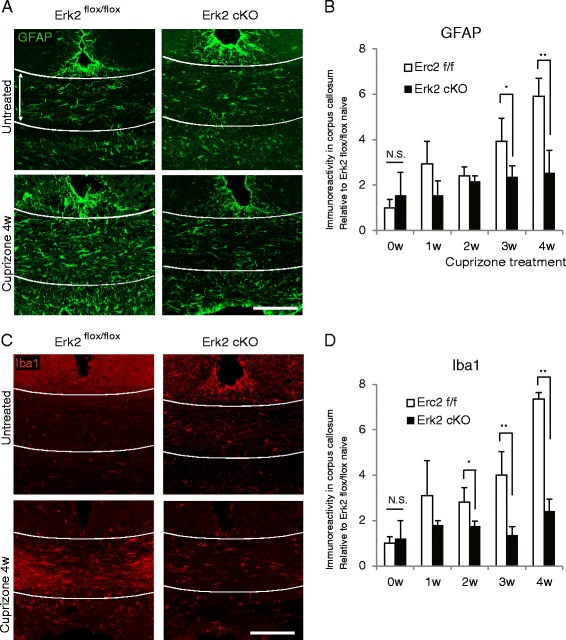
Deletion of Erk2 leads to lessened gliosis during cuprizone feeding. Histological samples of corpus callosum were obtained from either Erk2 cKO or Erk2 ^flox/flox^ mice before and 1, 2, 3, and 4 weeks after the start of cuprizone feeding. In Erk2 ^flox/flox^ mice, immunoreactivity for GFAP and Iba-1 is increased at 4 weeks compared to untreated (**a**, **c** *left*), while such changes are less prominent in Erk2 cKO mice (**a**, **c** *right*). **b**, **d** During 4 weeks of cuprizone feeding, the immunoreactivity for GFAP (**b**) and Iba-1 (**d**) in Erk2 ^flox/flox^ gradually increase, while the trend is less prominent in Erk2 cKO (**b**, **d**: *n* = 3 for each time point; Student’s *t* test; *p < 0.05; **p < 0.01). *Scale bars*: **a**, **c** 100 μm

In the process of cuprizone-induced demyelination, the increase in the expression of inflammatory mediators and gliosis proceeds simultaneously, resulting in augmented, non-specific noxious stimuli against white matter tissue. Because we observed a marked reduction of inflammatory mediators in Erk2 cKO at 4 weeks of cuprizone treatment, we further assessed the effect of Erk2 ablation on activation of astrocytes and microglia at that period. Immunohistological analyses revealed an increase in GFAP immunoreactivity in Erk2 ^flox/flox^ mice but no significant increase in Erk2 cKO mice after 4 weeks of cuprizone feeding (Fig. 8a, b). Then we evaluated the effect of Erk2 ablation on the accumulation and activation of microglia. As with the astrocytes, Iba-1 immunoreactivity was significantly lower in Erk2 cKO mice compared with Erk2 ^flox/flox^ mice after cuprizone feeding (Fig. 8c, d). These results indicate that Erk2 deletion abrogates inflammatory responses as well as accumulation of astrocytes and activated microglia. Taken together with the observation of white matter preservation in Erk2 cKO, we assume that the lack of inflammatory gliosis at 4 weeks provided a better environment for oligodendrocyte preservation in cuprizone-treated Erk2 cKO mice.

### Microglia-conditioned medium stimulated astrocytes to produce inflammation-related molecules

**Fig. 9:**
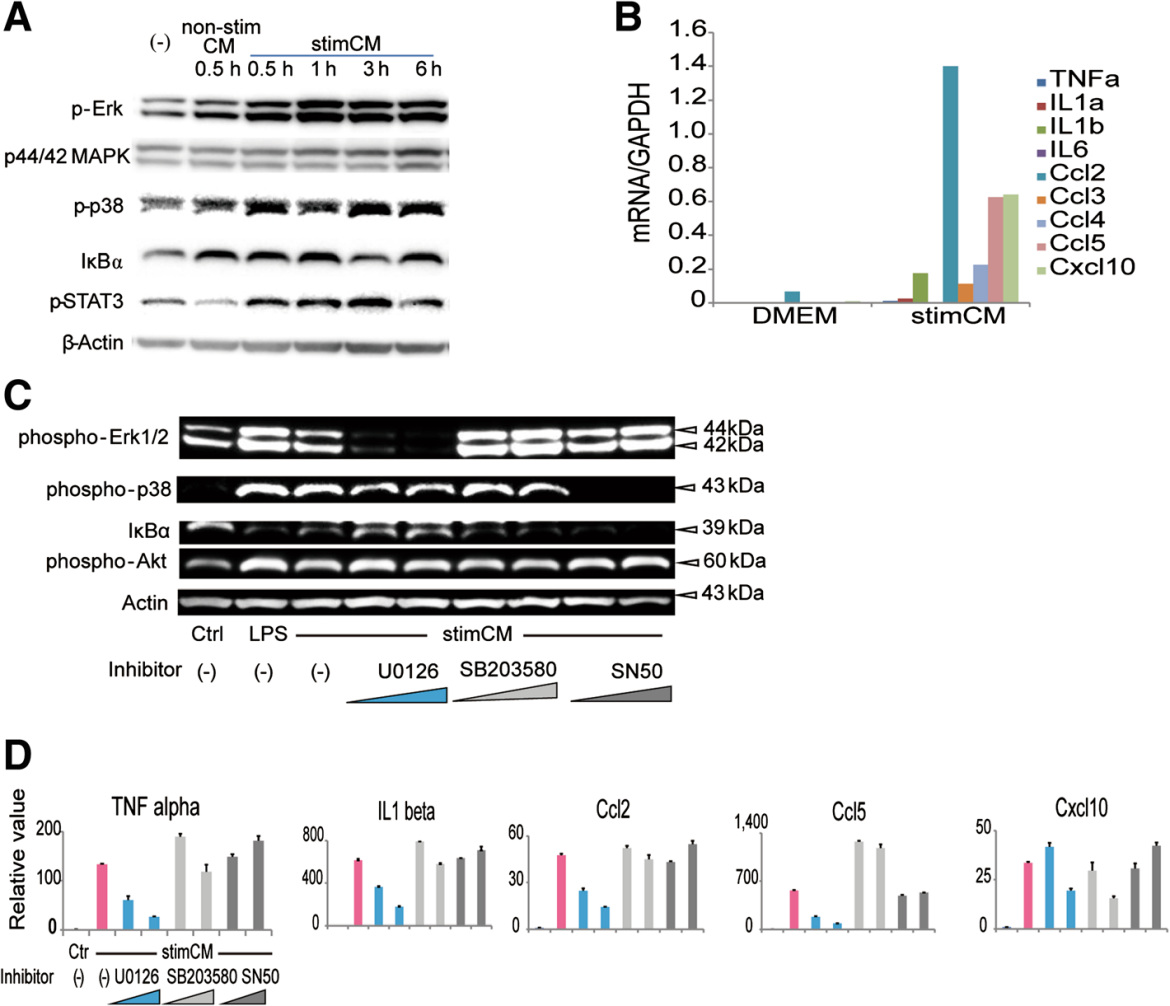
Cultured astrocytes produce inflammatory molecules in a Mek-Erk-dependent manner. A primary astrocyte culture is treated with medium conditioned with lipopolysaccharide (LPS)-stimulated microglia (stimCM) or non-stimulated microglia (non-stimCM). **a** Various signaling pathways including Erk1/2 are activated by stimCM. **b** Messenger RNA expression levels for cytokines and chemokines are elevated at 24 h after stimCM treatment. The results are shown as fold values relative to GAPDH. **c** The Mek inhibitor U0126 suppresses stimCM-induced Erk1/2 phosphorylation, while also modifying phosphorylation of p38 and degradation of Ikappa B-alpha. Other inhibitors, SB203580 and SN50, have minimal effects on phosphorylation of Erk1/2 (control: DMEM; LPS: DMEM supplemented with LPS). **d** Inhibition of Erk1/2 phosphorylation leads to lessened expression of TNF-alpha, IL-1beta, Ccl-2, Ccl-5, and Cxcl-10 in a dose-dependent manner (*n* = 3)

Our in vivo study revealed that “primary” oligodendrocyte cell damage and microglial activation were followed by “secondary” activation of astrocytes and that lack of Erk2 in astrocytes led to reduced expression of inflammation-related molecules, reduced gliosis, and ameliorated demyelination. In this context, we inferred that astrocytes received cell-to-cell signals from the primary responding cells, that is, microglia, which activated the formers’ intracellular Erk2 signaling pathways. Thus, to evaluate the contribution of the astrocytic Erk pathway to the production of inflammation-related molecules under pathological conditions, we established separate, purified cultures of microglia and astrocytes from neonatal cortices of C57BL/6 mice. Then, we treated the astrocyte culture with conditioned medium taken from the LPS-stimulated microglia culture (stimCM) or medium taken from non-stimulated microglia culture (non-stimCM). First, we examined the effect of stimCM on astrocytic intracellular signals in comparison to the non-stimCM control medium. Western blotting analyses showed that stimCM induces in astrocytes the phosphorylation of Erk1/2, p38 MAPK, and STAT3, concurrently with the degradation of IκBα (Fig. 9a). Then, quantitative RT-PCR analyses revealed that stimCM induces expression of inflammation-related molecules in astrocytes (Fig. 9b). To investigate the involvement of each signaling pathway, the Mek inhibitor U0126, the p38 MAPK inhibitor SB203580, the JNK inhibitor, and the NF-kB inhibitor SN50 were applied to the astrocyte culture individually. Among the various inhibitors, we found that the Mek inhibitor U0126 suppressed Erk phosphorylation more potently than did the others (Fig. 9c). Furthermore, the Mek inhibitor significantly reduced expression of TNF-alpha, IL-1beta, IL-6, Ccl-2, Ccl-3, and Ccl-5 in a dose-dependent manner (Fig. 9d). These results indicate that production of inflammation-related molecules by astrocytes depends on Erk1/2 signaling under pathological conditions.

### Production of inflammatory cytokines and chemokines was reduced in Erk2-ablated astrocytes

**Fig. 10:**
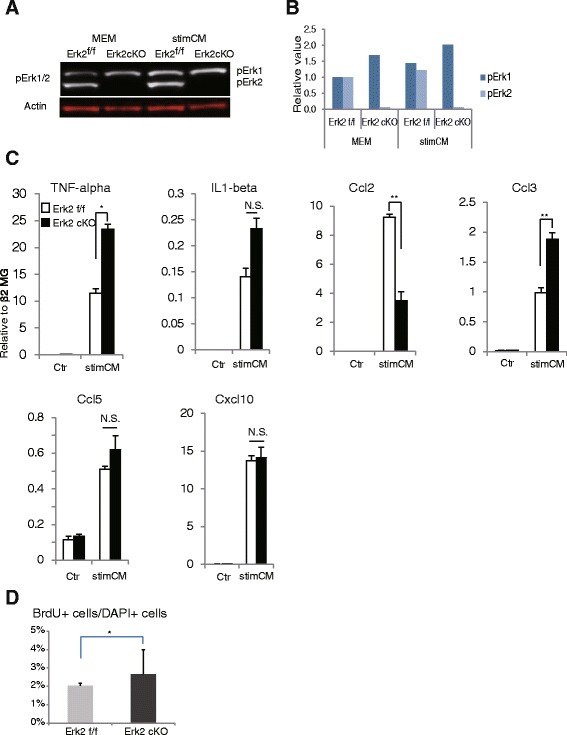
Inflammatory responses are reduced in Erk2-deficient astrocytes. Primary astrocyte cultures were obtained from either Erk2 cKO or Erk2 ^flox/flox^ mice. **a**, **b** Erk1/2 in Erk2 ^flox/flox^ astrocytes but only Erk1 in Erk2 cKO astrocytes are phosphorylated upon treating cultures with stimCM. **c** Astrocytes from Erk2 ^flox/flox^ mice express cytokines and chemokines after stimCM treatment. Among them, the expression of Ccl-2 is reduced in Erk2 cKO astrocytes at 3 h after stimCM treatment. *n* = 4; Student’s *t* test; *p < 0.05; **p < 0.01. **d** BrdU incorporation is examined under the same culture conditions. No significant difference is seen in the BrdU-positive/DAPI-positive ratio between Erk2 cKO and Erk2 ^flox/flox^ astrocytes. DAPI is a nuclear counterstain (*n* = 3; Student’s *t* test)

To evaluate the specific involvement of Erk2 in expression of inflammation-related molecules under pathological conditions, we established purified astrocyte cultures from embryonic cortices of Erk2 cKO or Erk2 ^flox/flox^ mice. We confirmed Erk2 deletion from the astrocytes by western blotting analysis (Fig. 10a, b). At 3 h after stimCM treatment, Erk2 deletion resulted in decreased expression of Ccl-2 and increased expression of TNF-alpha, IL-1beta, and Ccl-3 compared to Erk2 ^flox/flox^ astrocyte culture (Fig. 10c). In addition, to investigate the possible role of Erk2 in stimCM-induced proliferation, we quantified BrdU/GFAP double-positive cells in GFAP-positive astrocytes. There was no significant difference in the percentages of BrdU/GFAP double-positive cells between cultures (Fig. 10d). These findings indicate that Erk2 contributes to the expression of inflammation-related molecules but not to the proliferation of astrocytes under pathological conditions.

## Discussion

The results of our study demonstrated that (i) Erk1/2 is activated in astrocytes at 4 weeks after starting cuprizone feeding, when the increased expression of inflammation-related molecules is accompanied by progression of gliosis and demyelination in the corpus callosum and that (ii) deletion of Erk2 in astrocytes ameliorated these pathological reactions. The results with cultured astrocytes confirmed the role of astrocytic Erk2 in connecting stimuli from microglia to the production of inflammatory molecules from astrocytes. We infer that Erk2 within astrocytes plays a pivotal role in the progression of inflammation leading to demyelination.

### The role of astrocytes in inflammatory demyelination

Astrocytes maintain CNS homeostasis under physiological conditions, whereas they act as reactive astrocytes in the inflammatory environment. Gliosis, now defined as activation of astrocytes and microglia, is regarded not only as static scar tissue remaining after pathological events but also as an active component of the progression of diseases. In specimens from patients with multiple sclerosis, it has been reported that astrocytes surrounding demyelinated plaques express chemokines, which promote infiltration of inflammatory cells leading to exacerbated inflammation [28]. Because the astrocyte itself is not an immune cell, its role in inflammation is recognized in the context of cross talk with immune cells such as microglia. Several studies have reported interaction between microglia and astrocytes in the progression of inflammation [29, 30]. Although the precise pathogenic mechanism of the cuprizone model remains elusive, it has been perceived that cuprizone induces the primary event of oligodendrocyte cell death, which is followed by microglial accumulation [1, 23]. Our results reoreactivity characteristic of brain-resident microglia is increased at 1 week after cuprizone treatment and that these initial changes are similar between Erk2 cKO and control mice. In accordance with other reports, we observed increased immunoreactivity of both microglia (Iba-1+) and astrocytes (GFAP+) in control mice starting 3 weeks after the onset of cuprizone treatment [17, 31]. The progressive gliosis plays a part in removal of myelin-debris, as well as inducing further damages to oligodendrocytes as we observed in Fig. 4. We found that the progressive gliosis occurred to a much lesser extent in Erk2 cKO mice. Because nestin-driven deletion of Erk2 does not affect microglia and because Erk activation is confined to astrocytes, we infer that functional changes in astrocytes are responsible for the observed reduction of inflammatory molecule expression and ameliorated gliosis.

### Role of Erk2 in inflammation

As a member of the Erk family of intracellular signaling molecules, Erk2 has various roles depending on the context. In the CNS, it has been reported that Erk2 is involved in stem cell proliferation, oligodendrocyte differentiation, and extracellular matrix production by astrocytes [32, 33]. Several results from cultured astrocytes indicate the involvement of Erk1/2 in cytokine and chemokine production by these cells. Pedrazzi et al. reported that the nuclear factor high-mobility group box 1 (HMGB1) activates the Erk1/2 signaling pathway via the receptor for advanced glycation end products (RAGE), promoting expression of Cxcl-1, Cxcl-2, Ccl-2, and Ccl-5 [10]. In addition, Panenka et al. reported that activation of the P2X_7_ receptor on astrocytes induces expression of the chemokine monocyte chemoattractant protein-1 (MCP-1) in an Erk1/2-dependent manner [34]. Based on selective deletion of *ERK2*, our current results provide information about the specific role of Erk2. The conditioned medium from microglial culture induced several chemokines and cytokines. Among them, we found that expression of Ccl-2 is dependent on Erk2. The Erk2-dependent expression of Ccl-2 was recapitulated in vivo in the cuprizone model, in which Ccl-2 expression was also reduced in reactive gliosis tissue at 3 weeks after onset of the treatment. In accordance with the pro-inflammatory role of Ccl-2 [35–37], the reduced expression of Ccl-2 in vivo is accompanied by amelioration of microglia accumulation. It is noteworthy that in specimens from patients with multiple sclerosis, astrocytes surrounding demyelinating lesions express Ccl-2 [28]. Taken together, these data suggest that a lack of Erk2 in astrocytes results in a reduced production of chemokines, including at least Ccl-2, which further ameliorates the accumulation of inflammatory cells.

### Molecular targets for regulating inflammatory demyelination

Because prolonged inflammation within the CNS results in demyelination, regulating inflammation is a reasonable therapeutic strategy. In studies with the cuprizone model, various kinds of molecules, most of them related to inflammation, have been found to play pivotal roles in the inflammatory processes. Delayed demyelination has been reported in TNF-alpha, LT-alpha, LT-beta, and MIP1-alpha knockout mice [25, 38, 39, 27]. While these mice display degrees of demyelination similar to wild type at later experimental time points, our results shows that Erk2 cKO mice manifest milder demyelination all throughout the experimental period. Contrary to previous reports, Erk2 is not a humoral factor but an intracellular signaling molecule controlling many target genes. Although we identified Ccl-2 as a putative target of Erk2, it is possible that other molecules are also reduced in Erk2 cKO mice. Therefore, the prominent effect of Erk2 deletion in the cuprizone model indicates that intervention against molecules located relatively upstream in the inflammatory cascade may have relatively greater therapeutic potential.

### Application of the cuprizone model to CNS diseases

The cuprizone model is regarded as a model of multiple sclerosis [1, 18, 40]. Although there are several differences between the two pathologies, especially in the type of infiltrating inflammatory cells, both pathologies feature elevation of inflammatory molecules together with activation and proliferation of microglia and astrocytes, called gliosis. However, the full details of the hierarchy of inflammation, gliosis, and demyelination remain elusive. Our results show that inhibition of Erk2-dependent inflammatory-molecule production in astrocytes leads to reduced gliosis and demyelination, suggesting that activation of astrocytes is an upstream event occurring prior to the other two phenomena. Because gliosis is observed in many other disease models, the same intervention targeted to Erk2 functioning in astrocytes may have therapeutic effects in many diseases by reducing inflammation and gliosis formation. This is an issue for future research arising from our study.

### Limitations of the current model

In this study, we used a nestin promoter-driven Cre-expressing system, which removes the *ERK2* gene from all cell types in the neural lineage [20]. Although we observed that Erk activation is confined to astrocytes following cuprizone treatment, it is possible that functions of Erk2 in neurons and oligodendrocytes are also altered. Erk2 has been reported to play a part in the proliferation and maturation of oligodendrocytes and in the survival of neurons [32]. Because these functions of Erk2 may provide protective effects in models of inflammatory demyelination, we would anticipate that a lack of Erk2 in neurons and oligodendrocytes exacerbates tissue damage. However, our finding that Erk2 cKO mice manifested better tissue preservation than the control strain indicates that the function of Erk2 in neurons and oligodendrocytes has limited significance in this model.

As for cuprizone model, we adopted a 6-week feeding protocol while other groups use a 5-week feeding protocol [31]. We attempted to refer to previous works in gliosis after cuprizone treatment in which 6-week protocol were used [17, 22]. Because spontaneous remyelination starts from 5 weeks after treatment, the caution should be paid to compare our data of 5 weeks or later with data from 5-week-protocol experiment. However, because the progression of gliosis and inflammation becomes prominent by 4 weeks, we assume that our finding is also relevant in a 5-week feeding protocol.

It should also be noted that the type of inflammation varies between diseases [41]. Therefore, the role of Erk2 in astrocytes may differ between the cuprizone model and other demyelination models or human multiple sclerosis. Our finding is applicable when Erk2 is activated in astrocytes located within areas of reactive gliosis. Further study is required to compare different disease models and to provide a more generalized model of inflammatory gliosis.

## Conclusions

In conclusion, in the cuprizone-induced inflammatory demyelination model, astrocytes are activated together with microglia. Erk2 signaling contributes to the progression of the gliosis, partially by promoting cytokines and chemokines production from astrocytes. Ablating Erk2 signaling leads to both the reduction of gliosis and the preservation of oligodendrocytes Therefore, intervention to modify Erk2 functioning is a possible strategy for interfering with progressive inflammatory gliosis and can provide the insights needed to identify promising therapeutic molecular targets in inflammatory demyelinating diseases.

## Abbreviations

BrdU: Bromodeoxyuridine
Ccl: Chemokine (C-C motif) ligand
CNS: Central nervous system
Cxcl: Chemokine (C-X-C motif) ligand
Erk: Extracellular signal-regulated kinase
GAPDH: Glyceraldehyde-3-phosphate dehydrogenase
GFAP: Glial fibrillary acidic protein
GFP: Green fluorescent protein
HMGB: High-mobility group box
IL-1: Interleukin-1
JNK: c-Jun N-terminal kinase
LFB: Luxol fast blue
LPS: Lipopolysaccharide
MAG: Myelin-associated glycoprotein
MAPK: Mitogen-activated protein kinase
MBP: Myelin basic protein
MCP-1: Monocyte chemoattractant protein-1
MS: Multiple sclerosis
NF-kB: Nuclear factor-kappa b
PBS: Phosphate-buffered saline
PDL: Poly-d-lysine
TNF-alpha: Tumor necrosis factor alpha
STAT3: Signal transducer and activator of transcription 3
stimCM: Conditioned medium taken from the LPS-stimulated microglia culture
